# Impact of the inoculum size on the *in vivo* activity of the aztreonam-avibactam combination in a murine model of peritonitis due to *Escherichia coli* expressing CTX-M-15 and NDM-1

**DOI:** 10.1128/aac.01288-22

**Published:** 2024-12-19

**Authors:** Ariane Amoura, Laura Benchetrit, Sophie Magréault, Samuel Chosidow, Alice Le Menestrel, Vincent Jullien, Victoire de Lastours, Françoise Chau, Sara Dion, Laurent Massias, Bruno Fantin, Agnès Lefort

**Affiliations:** 1 Université Paris Cité, INSERM, IAME 54999 , Paris, France; 2 Universite Sorbonne Paris Nord, INSERM, IAME 167549 , Bobigny, France; 3 Laboratoire de Pharmacologie, Hôpital Jean Verdier 36808 , Bondy, France; 4 Service de Médecine Interne, Hôpital Beaujon 55100 , Clichy, France; 5 Laboratoire de toxicologie, Hôpital Bichat 55076 , Paris, France; Houston Methodist Hospital and Weill Cornell Medical College, Houston, Texas, USA

**Keywords:** aztreonam, avibactam, *Escherichia coli*, carbapenemase

## Abstract

The combination of aztreonam (ATM) and avibactam (AVI) is an attractive option to treat infections caused by extended spectrum β-lactamase plus NDM-1-producing *Enterobacteriaceae*. Since ATM activity was shown to be severely impacted by an increase in the inoculum size *in vitro*, we wondered whether ATM-AVI activity could be impaired in high-inoculum infections. We analyzed the impact of the inoculum size on ATM-AVI activity *in vitro* and in a murine model of peritonitis due to susceptible *Escherichia coli* CFT073-pTOPO and its isogenic derivatives producing NDM-1 (*E. coli* CFT073-NDM1) and CTX-M-15 plus NDM-1 (*E. coli* CFT073-CTXM15-NDM1). The impact of the inoculum size on bacterial morphology was studied by microscopic examination. *In vitro*, at standard (10^5^) inoculum, *E. coli* CFT073-CTXM15-NDM1 was resistant to ATM but susceptible to the ATM-AVI combination. At high (10^7^) inoculum, MICs of ATM alone and of the ATM-AVI combination reached >512 and 64 mg/L, respectively, against all tested strains. ATM led to bacterial filamentation when active against the bacteria, i.e., in monotherapy or in combination with AVI against susceptible *E. coli* CFT073-pTOPO and only in combination with AVI against *E. coli* CFT073-CTXM15-NDM1. *In vivo*, increase in the inoculum led to a drastic decrease in the activity of ATM alone against *E. coli* CFT073-pTOPO and ATM-AVI against *E. coli* CFT073-CTXM15-NDM1. Our results suggest a high *in vivo* impact of the inoculum increase on the activity of ATM alone against ATM-susceptible *E. coli* and of ATM-AVI against CTX-M-15 plus NDM-1 producing *E. coli*. Clinicians must be aware of the risk of failures when using ATM-AVI in high-inoculum infections.

## INTRODUCTION

The massive consumption of carbapenems to treat infections caused by extended spectrum β-lactamase-producing *Enterobacteriaceae* (ESBL-PE) has contributed to the emergence of carbapenemase-producing strains (CPE) whose rapid spread represents a worrisome threat worldwide (1). The major concern comes from metallo-β-lactamases (MBLs) which are endemic in many areas, such as India or Greece, because they confer resistance to nearly all available antibiotics, including novel β-lactams, leading to a therapeutic deadlock.

Aztreonam (ATM) is a natural β-lactam discovered in the 1980s. It is effective against gram-negative bacilli such as *Enterobacteriaceae* and *Pseudomonas aeruginosa* (2). The pharmacokinetic/pharmacodynamic (PK/PD) index that best predicts its antimicrobial activity is the percentage of time in a 24 hour period when the free drug concentration is above the minimal inhibitory concentration (MIC) (*%f*T_>MIC_) (3). An inventory has identified it as an old molecule worth studying thanks to its efficacy against MBLs (4). Indeed, ATM is the only β-lactam effective against MBLs. However, its efficacy against MBL-producing isolates is most of the time compromised by the co-production of serine-protease enzymes of Ambler’s β-lactamases classes A, C, and D, that hydrolyze ATM (3, 5). In addition, ATM activity was shown to be greatly impacted by the size of the inoculum, so-called “inoculum effect” *in vitro*, although the *in vivo* impact of this effect on *Enterobacteriaceae* bearing MBL remains unknown (6–8).

Avibactam (AVI) is a semisynthetic non-β-lactam β-lactamase inhibitor belonging to the diazabicyclooctanes (9). It is effective against serine-protease enzymes of Ambler’s class A, C, and D, by covalently acylating its serine target (10, 11). AVI alone does not display any intrinsic antimicrobial activity (12). PK/PD parameter that best correlates with the efficacy of AVI in combination with ATM is the percentage of time in a 24 hour period when the free drug concentration is above the critical threshold of 2.5 mg/L (%*f*T_CT>2.5 mg/L_) (9, 13).

The idea of combining ATM and AVI was hence put forward as AVI is stable against ESBL-PE while ATM is effective against MBLs such as NDM-1 enzyme (14). The *in vitro* efficacy of ATM-AVI has been demonstrated (14) but an inoculum effect of the combination was evidenced (15–18). A few pharmacokinetic (PK)/pharmacodynamic (PD) studies on murine models supported the benefit of the combination (13, 19). Some patients have already been successfully treated by ATM and ceftazidime-AVI (CZA) because ATM-AVI was not available for human use (20–23). Recent phase 2a and phase 3 studies investigated PDs and safety of ATM-AVI in patients with various infections (24, 25). Thus, IDSA recommended using CZA and ATM to treat MBL-producing *Enterobacteriaceae* although extra data are necessary especially regarding the *in vivo* impact of the inoculum effect (26). The ATM-AVI (1.5/0.5) combination has since been approved in Europe.

Therefore, the aims of our study were to assess the efficacy of ATM-AVI combination against an NDM-1 plus CTX-M-15 producing *Escherichia coli* strain, by comparison to its activity against β-lactam susceptible parental strain, in a murine model of peritonitis at standard and high inocula.

## MATERIALS AND METHODS

### Bacterial strains and plasmids

To conduct our experiments, three isogenic strains were constructed. Details of construction have already been reported (27). Briefly, susceptible uropathogenic *E. coli* CFT-073 (O6:K2:H1) (28) was used as recipient for the plasmid pCR-Blunt II-TOPO (Life Technologies, Saint-Aubin, France) carrying a kanamycin resistance gene. Regions corresponding to *bla*_NDM-1_ and *bla*_CTX-M-15_ were cloned in the pCR-Blunt II-TOPO resulting in plasmids pTOPO-NDM1 and pTOPO-CTXM15-NDM1. These plasmids were introduced by electrotransformation into *E. coli* CFT073, as described previously, resulting in *E. coli* CFT073-pTOPO, *E. coli* CFT073-NDM1, and *E. coli* CFT073-CTXM15-NDM1. Strains and subcultures were grown in Mueller Hinton (MH) broth using kanamycine (400 mg/L) to avoid all plasmid loses.

### Antimicrobial agents

Antibiotics used were kanamycin (Sigma-Aldrich, Saint-Quentin-Fallavier, France), ATM (Sigma-Aldrich, Saint-Quentin-Fallavier, France, for *in vitro* experiments; Sanofi, Gentilly, France, for *in vivo* experiments), AVI (Sigma-Aldrich, Saint-Quentin-Fallavier, France, for *in vitro* experiments; MedChemEpress, Sollentuna, Sweden, for *in vivo* experiments), and imipenem (IPM) (Arrow, Lyon, France).

### *In vitro* experiments

#### Growth rate and fitness

Growth curves in Luria-Bertani medium were performed by measuring the OD at 600 nm every 5 minutes for 24 hours at 37°C using an automatic spectrophotometer (Tecan Infinite F200PRO, Männedorf, Switzerland). Results were analyzed using R software which provided maximum growth rates (MGR) used to compare bacterial fitness (29).

#### MICs

MICs of ATM, AVI, ATM in the presence of a fixed concentration of 4 mg/L AVI (as recommended by the European Committee on Antimicrobial Susceptibility Testing [EUCAST] for ATM plus AVI MIC determination), and IPM were determined by the microdilution method in MH broth in accordance with EUCAST (30).

To evaluate the impact of the inoculum on antibiotic activity, MICs were performed at standard (5 × 10^5^ CFU/mL) and high (5 × 10^7^ CFU/mL) inocula. To analyze whether the results were dependent on the method used for MIC determination, MICs of ATM against *E. coli* CFT073-pTOPO were also determined by the agar dilution method of Steers et al., at standard (5 × 10^5^ CFU per spot) and high (5 × 10^7^ CFU per spot) inocula (31).

The impact of antibiotic exposure on bacterial morphology was analyzed before and after 24-hour antibiotic exposure using an optical microscope (Zeiss, Oberkochen, Germany) using a 100× objective after Gram staining.

All experiments were performed at least three times.

#### Time kill curves at standard and high inocula and selection of mutants

For time kill curves, a culture of each strain was grown overnight in MH containing kanamycin (400 mg/L). The culture was diluted 1/1,000 and incubated for 4.5 hours to obtain an exponential phase culture of 10^7^ to 10^8^ CFU/mL for the high-inoculum assay. This culture was diluted 1/100 for the standard inoculum assay. ATM was added at 40 mg/L, four times the residual free drug concentration in infected mice, and AVI at 4 mg/L. CFU were enumerated by serial dilutions after centrifugation and resuspension of the pellets to avoid carry-over effects at 1, 3, and 24 hours. After 24 hours, the cultures were also plated on plates containing four times the MIC of ATM and 4 mg/mL AVI to look for resistant mutants. The detection limit was 1 colony per 100 µL.

### *In vivo* studies

#### Animal care

Swiss ICR-strain female mice weighing approximately 25 g were used. Animals were housed in regulation cages and given free access to food and water. Overall, 277 mice were used for experiments.

#### Antibiotic PKs

Antibiotics were injected subcutaneously into previously infected mice using the CFT073-pTOPO strain. ATM 100 mg/kg and AVI 100 mg/kg were administered separately or together in order to check for the absence of drug-drug interaction. Blood samples were obtained by intracardiac puncture from previously anesthetized mice (three mice for each point), 15, 30, 60, and 120 minutes after injection of ATM or AVI or the combination.

Therapeutic regimens were chosen to achieve in serum a percentage of time during which the free drug concentrations exceeded the MIC (*%f*T_>MIC_) for ATM (considering MIC determined in standard conditions, according to the EUCAST recommendations, with a breakpoint of 1 mg/L for susceptibility), and ≥2.5 mg/L (%*f*T_CT>2.5 mg/L_) for AVI, close to those obtained in humans with recommended dosages. The assays carried out by the laboratory yielded total fractions. We deduced the free fractions from protein binding in mice, assumed to be 43.5% for ATM and 10% for AVI (19).

The free area under the curve (AUC) of ATM and AVI was first calculated by non-compartmental analysis after a single dose of 100 mg/kg in each mouse when the drugs were administered alone or in combination. Plasma concentrations measured in mice were then pooled and modeled to determine primary PK parameters for ATM and AVI (clearance and volume of distribution) using the Stochastic Approximation Expectation Minimization algorithm by Monolix v.2023R1 (Lixoft, Orsay, France; http://www. lixoft.com).

To describe PK profiles in humans, typical PK parameters of ATM and AVI were used (32). Humans and mice free AUC were calculated over a 24-hour period at a steady state for a dose of 1.5 g ATM and 500 mg AVI every 6 hours (1-hour infusion) in humans and the selected dose regimen of ATM and AVI in mice. Free AUC were determined using the following formula: *f*AUC = fu × dose/clearance, with fu being the free fraction of the drug.

Simulations were then carried out to estimate the PK/PD target attainment using Simulix v.2023R1 (Lixoft, Orsay, France; http://www. lixoft.com). The previously estimated PK parameters of ATM and AVI were used for mice. For humans, 200 subjects were simulated using typical PK parameters and inter-subject variability (32). Concentrations were simulated every 0.05 hours, and the %fT_>MIC_ for ATM and %fT_CT>2.5 mg/L_ for AVI were automatically calculated.

IPM was used for comparison because its activity is only slightly altered by the inoculum effect (33). *In vivo*, a therapeutic regimen of 100 mg/kg/4 hours of IPM was chosen according to previous results of our team (27).

#### Murine model of peritonitis

The murine model of peritonitis was used as previously described (34). Briefly, overnight cultures of each strain were mixed with porcine mucin 10% (Sigma-Aldrich, Saint-Quentin-Fallavier, France) to enhance bacterial infectivity. Mice were inoculated with an intraperitoneal injection of 250 µL of bacteria/mucin mix corresponding to a final inoculum of 10^6^ CFU (standard inoculum) of 10^8^ CFU (high inoculum). Two hours after inoculation, groups of 5 to 18 mice were treated with ATM, AVI, ATM plus AVI, or IPM for 24 hours. For each strain and inoculum, at least three mice per group were sacrificed 2 hours after inoculation to determine bacterial load (“start-of-treatment” [SOT] controls). End of treatment (EOT) controls were sacrificed 28 hours after infection to determine the bacterial load in untreated animals. In order to avoid animal suffering, animal well-being was evaluated every 4 hours and then hourly if necessary according to protocol, and mice were sacrificed if critical score was achieved (35). Sacrifice was done by intraperitoneal injection of 400 mg/kg of sodium thiopental. Mice that survived during the whole duration of treatment were sacrificed 4 hours after the last antibiotic injection.

After death, the spleen was extracted and homogenized in 1 mL of sterile saline solution. Samples were plated onto agar containing kanamycin for quantitative culture. Results were expressed as log_10_ CFU/g for the spleen. The detection limit was 1 log_10_/g.

### Statistical analysis

Results were expressed as median [minimum-maximum] for continuous variables. MGR and log_10_ CFU/g in spleen were compared for the different groups by the Mann-Whitney U-test or the Kruskal-Wallis test when appropriate. Death rate (mice sacrificed before the end of protocol) in the different groups and AUC of the different PK assays were compared by Fisher’s exact test. A *P* < 0.05 was considered significant.

## RESULTS

### *In vitro* studies

#### Growth rate and fitness

MGRs were 1.09 hours^−1^ [0.99–1.22], 0.93 hours^−1^ [0.89–1.15], and 0.82 hours^−1^ [0.74–0.90] for *E. coli* CFT073-pTOPO, *E. coli* CFT073-NDM1, and *E. coli* CFT073-CTXM15-NDM1, respectively. MGR was significantly lower for the two strains carrying the NDM-1 plasmid compared with *E. coli* CFT073-pTOPO (*P* < 0.001). Also, MGR of *E. coli* CFT073-CTXM15-NDM1 was significantly lower than that of *E. coli* CFT073-NDM1 (*P* < 0.001).

#### MICs

MICs of ATM, AVI, ATM-AVI (4 mg/L), and IPM against each strain at standard and high inocula are shown in Table 1.

**TABLE 1:** MICs of ATM, AVI, ATM with a fixed concentration of AVI 4 mg/L, and IPM determined by the broth microdilution method against *E. coli* strains at standard (10^5^ CFU/mL) and high (10^7^ CFU/mL) inocula

*E. coli* strain	MIC (mg/L) according to inoculum size (CFU/mL)							
	ATM		AVI		ATM-AVI^*a*^		IPM	
	10^5^	10^7^	10^5^	10^7^	10^5^	10^7^	10^5^	10^7^
CFT073-pTOPO	0.0625	>512	256	>512	0.0625	64	0.5	1
CFT073-NDM1	0.0625	>512	256	512	0.031	64	256	>1,024
CFT073-CTXM15-NDM1	256	>512	256	>512	0.031	64	64	>1,024

^*a*^ Fixed 4 mg/L concentration. ATM, aztreonam; AVI, avibactam; IPM, imipenem.

At standard (10^5^) inoculum, ATM was highly active against *E. coli* CFT073-pTOPO and *E. coli* CFT073-NDM1. CFT073-CTXM15-NDM1 was resistant to ATM but its activity was completely restored by the adjunction of AVI 4 mg/L. MICs of AVI against all tested strains were 256 mg/L, indicating the absence of significant intrinsic activity of AVI alone against these strains.

A major *in vitro* inoculum effect of ATM was evidenced, with MICs of ATM alone and of the ATM-AVI combination reaching >512 and 64 mg/L, respectively, at high (10^7^) inoculum, against all tested strains.

A major inoculum effect was also observed when MICs of ATM against *E. coli* CFT073-pTOPO were measured by the method of Steers et al. (31). Indeed, the MIC of ATM increased from 0.125 to 64 mg/L when increasing the inoculum from 10^5^ to 10^7^ CFU per spot, indicating that the observed inoculum effect was independent of the method used.

#### Impact of antibiotic exposure on bacterial morphology

**Fig 1:**
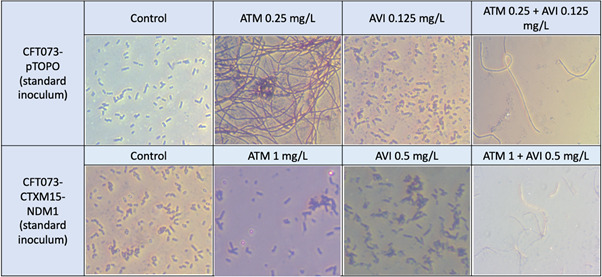
Gram stain morphology of *E. coli* CFT073-pTOPO and *E. coli* CFT073-CTXM15-NDM1 after 24 hours of incubation with ATM, AVI, or ATM-AVI or without antibiotic (control).

Microscopic examination of bacteria recovered after 24 hours of incubation with ATM, AVI and the combination at standard inoculum is shown in Fig. 1. Exposure of *E. coli* CFT073-pTOPO to ATM either alone or combined with AVI and of *E. coli* CFT073-CTXM15-NDM1 to ATM-AVI resulted in bacterial filamentation. Exposure of *E. coli* CFT073-CTXM15-NDM1 to ATM alone did not result in bacterial filamentation. AVI alone had no effect on bacterial morphology. These results were the same with a standard or high inoculum (data for high inoculum not shown). Thus, ATM led to bacterial filamentation when active against the bacteria, i.e., in monotherapy or in combination with AVI against susceptible *E. coli* CFT073-pTOPO and only in combination with AVI against *E. coli* CFT073-CTXM15-NDM1.

#### In vitro bactericidal activity of antibiotics and selection of mutants

**Fig 2:**
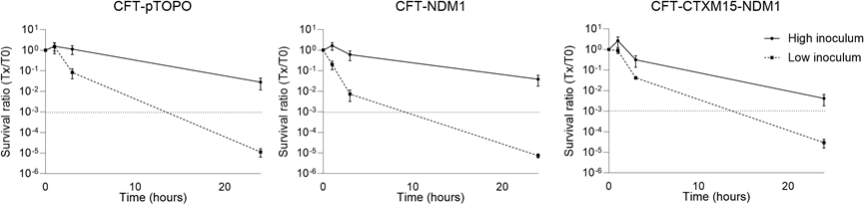
Time kill curves of ATM 40 mg/L and AVI 4 mg/L against *E. coli* CFT073-pTOPO, *E. coli* CFT073-NDM1, and *E. coli* CFT073-CTXM15-NDM1. Results are expressed as survival ratios (Tx/T0). High inoculum was between 5 × 10^7^ and 2 × 10^8^ CFU/mL for *E. coli* CFT073-pTOPO, 1 × 10^8^ and 2 × 10^8^ CFU/mL for *E. coli* CFT073-NDM1, and 2 × 10^7^ and 7 × 10^7^ CFU/mL for *E. coli* CFT073-CTXM15-NDM1. The standard inoculum was 100 times lower than the high inoculum.

Time kill curves of ATM 40 mg/L combined with AVI 4 mg/L against the three isolates at standard and high inocula are shown in Fig. 2. The concentration of ATM was chosen as it is equal to four times the free residual concentration in the infected mice treated with a single dose of 100 mg/kg ATM and thus seemed clinically relevant. The combination was bactericidal at 24 hours against the three isolates when a standard inoculum was used, but no bactericidal effect was observed at high inoculum. No mutants resistant to the combination were observed at 24 hours at low or high inoculum.

### *In vivo* studies

#### Therapeutic regimen

**Fig 3:**
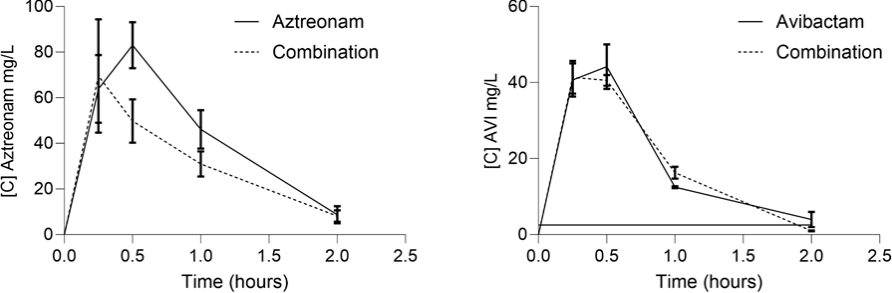
PKs of ATM and AVI in infected mice. Female Swiss mice weighing 25 g were infected with an intraperitoneal injection of 10^6^ CFU of *E. coli* CFT073-pTOPO and treated 2 hours after infection with 100 mg/kg ATM or 100 mg/kg AVI or a combination of both drugs. Mice were sacrificed 15, 30, 60, and 120 minutes after treatment, and intracardiac puncture was performed. Graph represents free serum dose high-performance liquid chromatography (HPLC) over time (*n* = 3 to 6 mice per time point).

There was no significant difference in dosages when drugs were administered separately or in combination (Fig. 3). Indeed, free AUC after administration of a single dose was not statistically different between ATM alone and in combination (86.1 and 63.3, respectively, *P* = 0.31) nor between AVI alone and in combination (38.3 and 38.0, *P* = 0.95), which allowed PK modeling to be performed on the pooled dataset.

**Fig 4:**
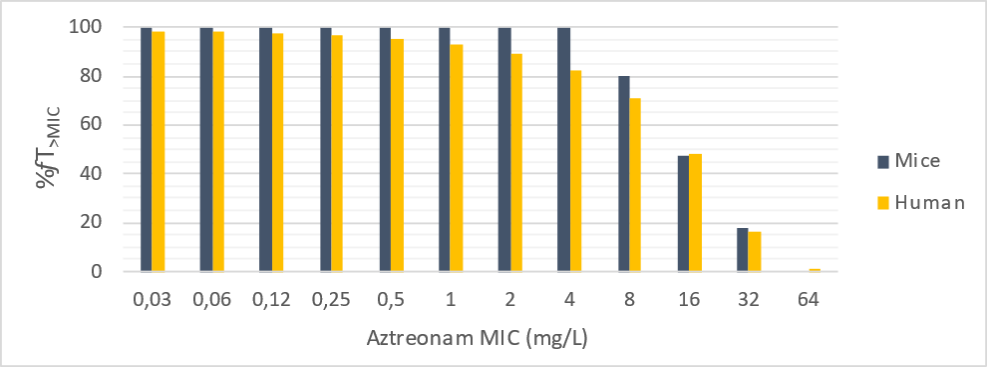
%fT_>MIC_ in humans and mice for different ATM MIC values.

A one-compartment model with zero-order absorption and linear elimination best described the data of ATM and AVI in mice. A proportional model and a mixed error model were used for ATM and AVI, respectively. PK parameters and their relative standard errors are reported in Table S1. Goodness-of-fit plots did not show any bias (Fig. S1). In humans, data were simulated using a one-compartment model with linear elimination. %fT_>MIC_ in humans and mice for different MIC values of ATM are presented in Fig. 4.

Using a regimen of 100 mg/kg every 2 hours for ATM and AVI used in combination, the percentages of *f*T_>MIC_ (ATM) and *f*T_CT>2.5 mg/L_ (AVI) were 100% and 72.9%, close to those obtained in humans at dosages recommended for severe infections (98.0% and 70.3%, respectively) (13) (Table 2).

**TABLE 2:** Seric concentrations (total), proportions of time during which free serum concentrations exceed the MIC (%fT_>MIC_) for ATM and the critical threshold of 2.5 mg/L (%fT_CT>2.5 mg/L_) for AVI, and steady-state free AUC_24h_ according to therapeutic regimens used in humans and in mice infected with CFT073-pTOPO

Species	Antibiotic	ATM PKs			AVI PKs		
		Peak (mg/L)	*f*T_>MIC_ (%)^*a*^	*f*AUC_24h_ (mg.h/L)	Peak (mg/L)	*f*T_CT>2.5 mg/L_ (%)	*f*AUC_24h_ (mg.h/L)
Mice	ATM 100 mg/kg	158 ± 138	100%	579			
	AVI 100 mg/kg				45 ± 8	72.9%	221
Humans	ATM 1.5 g q6h	102.9 ± 31.3	98.0%	530			
	AVI 500 mg q6h				19.3 ± 6.3	70.3%	157

^*a*^ *E. coli* CFT073-pTOPO and *E. coli* CFT073-CTXM15-NDM1 standard determination MICs.

#### Peritonitis

**Fig 5:**
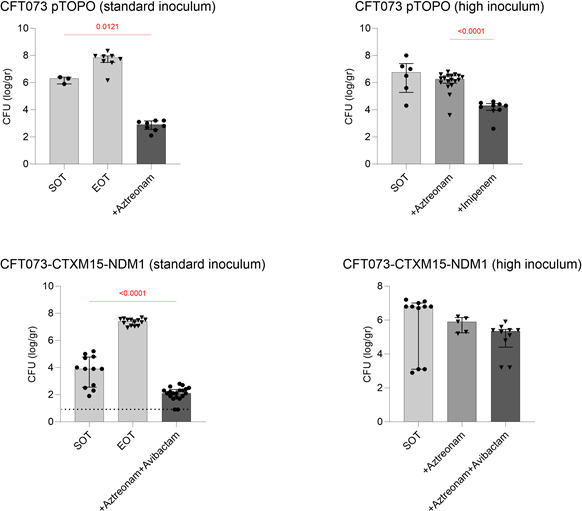
Efficacy of ATM or the combination of ATM and AVI in a mouse model of peritonitis at standard and high inocula. Five-week-old Swiss mice were infected with 10^6^ (standard inoculum) or 10^8^ (high inoculum) of *E. coli* CFT073-pTOPO and *E. coli* CFT073-CTXM15-NDM1. The SOT controls were killed 2 hours after infection. EOT controls were sacrificed 28 hours after infection. Treatment was started 2 hours after infection and continued for 24 hours. Bacterial counts are expressed as log_10_ CFU/g of spleen. Each point represents one mouse. A circle is a mouse alive at the endpoint, and a triangle is a mouse that died before the endpoint. Histograms are mean values, and bars are standard errors of the mean. The detection limit is one colony per 100 µL and is represented by a dotted line.

The efficacy of ATM and ATM-AVI in animals infected with *E. coli* CFT073-pTOPO and *E. coli* CFT073-CTXM15-NDM1, at standard and high inocula, is shown in Fig. 5. Full results are provided in Table S2.

Using a standard inoculum, AVI alone had no activity against *E. coli* CFT073-CTXM15-NDM1 (death of 5/5 animals before EOT, median [range] log10 CFU/g of spleen after treatment, 4.4 [4.2–4.8], *P* = 0.29 versus SOT controls). Based on this result and for ethical reasons, AVI alone was not tested at higher inoculum and/or against *E. coli* CFT073-pTOPO, since it was supposed to be inactive.

At standard inoculum, ATM alone was active in animals infected with *E. coli* CFT073-pTOPO in terms of both animal survival and bacterial decrease in spleens. In animals infected with *E. coli* CFT073-CTXM15-NDM1, ATM alone had no significant activity in terms of spleen bacterial decrease by comparison to controls but led to bacterial survival; the combination of ATM and AVI displayed significant activity, with survival of 100% of animals and a significant decrease in bacterial counts as compared with controls.

An increase in the inoculum led to a drastic decrease in the antibiotic activity of ATM alone against *E. coli* CFT073-pTOPO and of ATM-AVI against *E. coli* CFT073-CTXM15-NDM1. Indeed, using the same antibiotic regimens, all animals died and no significant reduction in spleen bacterial counts, as compared with controls, could be observed. At high inoculum, IPM was significantly active against *E. coli* CFT073-pTOPO.

Altogether, our results suggest a high impact of the inoculum increase on the activity of ATM against *E. coli* CFT073-pTOPO and of the ATM-AVI combination against *E. coli* CFT073-CTXM15-NDM1 *in vivo*.

## DISCUSSION

In the present study, we evidenced the *in vitro* activity of the ATM-AVI combination against CTX-M-15 plus NDM-1 producing *E. coli* in standard experimental conditions. ATM alone was effective against *E. coli* CFT073-pTOPO and *E. coli* CFT073-NDM1 but inactive in the presence of the CTX-M-15 enzyme, as previously demonstrated (14). AVI alone had no antibacterial activity against the three studied strains; however, its presence was essential to restore the full activity of ATM against *E. coli* CFT073-CTXM15-NDM1(12).

Exposition to a high bacterial inoculum led to a significant increase of the MICs of ATM rendering previously susceptible strains resistant to ATM according to EUCAST breakpoints (30). This inoculum effect was independent of the method used for MIC determination. The explanations for the inoculum effect are not clearly defined, and many mechanisms may probably coexist (36). An explanation often put forward is the high amount of production of β-lactamases at high inoculum that may overwhelm the amount of β-lactam (without and with a β-lactamase inhibitor); however, this mechanism is probably not the only one (37) and does not explain the inoculum effect we observed with susceptible *E. coli* CFT073-pTOPO (34, 38). Another explanation may be the selection of pre-existing subpopulations resistant to ATM; of note, no mutants were retrieved at 24 hours when time kill curves were performed at high inoculum. The main explanation we propose for this loss of activity at high inoculum may be the filamentous growth of bacteria we observed, as others (33), in the presence of ATM. Indeed, ATM binds exclusively to penicillin-binding protein 3 (PBP3), which plays an important role in peptidoglycan synthesis during the division process (39). When PBP3 is inhibited, the segmentation of bacteria into bacilli is prevented, resulting in a filamentous growth of the strains (40, 41). Accordingly, we observed a bacterial filamentation when ATM was incubated with bacteria, either alone or combined with AVI, provided that the bacteria were susceptible to the antibiotic used (ATM alone against *E. coli* CFT073-pTOPO, ATM-AVI combination against *E. coli* CFT073-CTXM15-NDM1). By contrast, no filamentation was observed when ATM was incubated with ATM-resistant *E. coli* CFT073-CTXM15-NDM1. Our results suggest that the inoculum effect may result from a change in the metabolic state of the bacteria, with a filamentation induced by ATM, which increases the bacterial biomass and density. At high inoculum, this increase in biomass is more noticeable than that at low inoculum; it alters the susceptibility of these bacteria to ATM with or without AVI, which fails to reduce the number of viable organisms. Our results suggest that MIC determination, using the recommended EUCAST method with a standard inoculum of 5 × 10^5^ bacteria, could be inappropriate to predict ATM or ATM-AVI activity in high-inoculum situations.

It is important to assess the impact of the *in vitro* inoculum effect on the *in vivo* efficiency of antibiotic treatment, particularly in case of infection with large bacterial burdens, such as abscesses, necrotizing fasciitis, or severe ventilator-associated pneumonia (42, 43), and the mouse model of peritonitis is a relevant model in this situation (34). In fact, an *in vitro* inoculum effect doesn’t always lead to treatment failure *in vivo*. For example, using the same *in vivo* model, our team showed that despite a high-inoculum effect *in vitro*, cefiderocol remained effective in a high-inoculum peritonitis infection (44). In our *in vivo* experiment, ATM permitted a significant decrease in bacterial count and the survival of all mice infected when animals were infected with a “standard” inoculum (inoculation of 10^6^ CFU) of *E. coli* CFT073-pTOPO. Similarly, ATM plus AVI was effective in mice infected with a “standard inoculum” of *E. coli* CFT073-CTXM15-NDM1. In the presence of a high inoculum (inoculation of 10^8^ CFU to animals), ATM against *E. coli* CFT073-pTOPO and ATM-AVI against *E. coli* CFT073-CTXM15-NDM1 were ineffective. Indeed, none of the mice in these groups survived until the end of protocol.

Therapeutic regimens used in mice led to concentrations close to those recommended in humans. For ATM, the %*f*T_>MIC_ were very similar (100% vs. 98% in mice and humans, respectively), as is the total exposure represented by the *f*AUC (579 vs. 530 mg.hours/L). For AVI, the *f*AUC in mice was slightly higher than that predicted in humans (221 vs. 157 mg.hours/L). However, we believe that the efficacy of this molecule is related to the % *f*T_CT>2.5 mg/L_, and this time is similar in mice and humans (72.9% vs. 70.3%, respectively). It should also be noted that the predicted human concentrations in this study are similar to those previously described in the literature (13, 24).

In accordance with the *in vitro* results, the main hypothesis to explain the *in vivo* results could be a decreased ATM efficacy in the presence of a high inoculum. This hypothesis is backed up by the maintained activity of IPM against *E. coli* CFT073-pTOPO at high inoculum. Indeed, it was previously shown that IPM activity is poorly affected by the inoculum effect (33). Unlike ATM, IPM mainly binds to PBP2 (45). Thus, treatment with IPM does not lead to bacterial filamentation but to spheroplast formation (46). The difference in the target protein between ATM and IPM may be a possible explanation for the greater inoculum effect we observed with ATM than with IPM. The impact of the inoculum on ATM has already been suggested by Soriano et al., reporting the need to drastically increase the dosage of antibiotics with a pronounced inoculum effect, such as ATM, to decrease mortality in bacteriemic rats (6). In the REJUVENATE study, a phase 2a open study evaluating the PKs and safety of ATM plus AVI for the treatment of complicated intraabdominal infections, which presents the largest prospective cohort of patients treated with ATM-AVI, with 58.8% clinical cure rates at the test-of-cure visit, all patients had a surgical procedure for source control and peritoneal lavage responsible for a decreased inoculum (24).

In conclusion, we show here the *in vivo* impact of the inoculum on the activity of ATM plus AVI against MBL-producing *Enterobacteriaceae*. ATM-AVI remains a promising option to treat infections caused by MBL-producing *Enterobacteriaceae*, but treatment failures leading to deaths or recurrences were reported (20, 22). Our study suggests the implication of the inoculum effect in the onset of failures, although it cannot be demonstrated in clinical practice. Clinicians must be aware of the risk of failures when using ATM-AVI in high-inoculum infections.

## SUPPLEMENTAL MATERIAL

Supplemental material aac.01288-22-s0001.docx Tables S1 and S2; Figure S1.

ASM does not own the copyrights to Supplemental Material that may be linked to, or accessed through, an article. The authors have granted ASM a non-exclusive, world-wide license to publish the Supplemental Material files. Please contact the corresponding author directly for reuse.
